# Gene expression of WWOX, FHIT and p73 in acute lymphoblastic leukemia

**DOI:** 10.3892/ol.2013.1514

**Published:** 2013-08-05

**Authors:** XU CHEN, HUI ZHANG, PING LI, ZHENG YANG, LINGYAN QIN, WUNING MO

**Affiliations:** Department of Clinical Laboratory, First Affiliated Hospital of Guangxi Medical University, Nanning, Guangxi 530021, P.R. China

**Keywords:** WWOX, FHIT, p73, acute lymphoblastic leukemia, mRNA expression, methylation

## Abstract

The aim of the present study was to analyze the expression of WW-domain oxidoreductase (WWOX), fragile histidine triad (FHIT) and p73 in acute lymphoblastic leukemia (ALL). Samples from 122 ALL patients and 35 non-ALL control patients were collected in this study. RT-PCR was performed to detect the mRNA expression of WWOX, FHIT and p73. The methylation status of the WWOX promoter region, FHIT promoter region and the first exon region of p73 were also analyzed using the methylation-specific PCR method. The mRNA expression of WWOX, FHIT and p73 was significantly lower in the ALL samples compared with the controls (48.2, 42.9 and 55.4%, respectively). By contrast, the methylation frequency of WWOX, FHIT and p73 was significantly higher in the ALL samples compared with the controls (44.6, 46.4 and 37.5%, respectively). The mRNA expression of these three genes was inversely correlated with the methylation frequency in the ALL samples (correlation coefficients, −0.661, −0.685 and −0.536 for WWOX, FHIT and p73, respectively). Moreover, the mRNA expression of WWOX was positively correlated with that of FHIT and p73 (correlation coefficients, 0.569 and 0.556, respectively). However, the methylation status of WWOX had no correlation with that of FHIT or p73. It was concluded that the high methylation status of WWOX, FHIT and p73 may lead to the inactivation of expression and the silencing of these genes, promoting the occurrence and development of ALL. The determination of the mRNA expression and methylation status of WWOX, FHIT and p73 may aid in the development of treatment approaches for ALL.

## Introduction

Acute lymphoblastic leukemia (ALL) comprises a heterogeneous group of lymphoid malignancies, which affect 1–4.75 individuals per 100,000 worldwide (1–2). Certain Western countries, including Italy, the United States, Switzerland and Costa Rica account for the highest incidence of ALL (3). Of all the leukemia cases in children, ~80% are ALL. The incidence rate has increased remarkably, with an annual percentage change of 0.8% between 1975 and 2006 (4,5). In children aged 1–4 years, the annual percentage change reaches 1.2% (3). The high mortality and morbidity rates considerably affect living standards and survival quality. However, at present, the exact etiology of ALL remains unknown, although the majority of studies have considered it to be associated with infection, radiation, chemical and genetic factors (3,4).

Over 80 common fragile sites have been identified in the human genome database (6). Common fragile sites are chromosomal regions that are observed in metaphase chromosomes. The genes in these regions are more susceptible to breakage, rearrangements and deletions compared with other genes (7,8). Studies have demonstrated that the genes associated with common fragile sites have important roles in carcinogenesis (7,8).

Carcinogenesis is a complicated multipathway processes involving genetic alterations, including the inactivation of tumor suppressor genes. Fragile histidine triad (FHIT) and WW-domain oxidoreductase (WWOX) are tumor suppressor genes that encompass the two most active common fragile sites. Since FHIT and WWOX were first identified by Ohta *et al*(9) and Bednarek *et al*(10), respectively, the loss or reduction of FHIT and WWOX expression has been shown to be an important step in the initiation of tumorigenesis in a variety of tumors, including those in the breasts, lungs, esophagus, kidneys and cervix (11–16). Despite the clear mechanism, evidence has demonstrated that possible synergy may exist between FHIT and WWOX in the pathogenesis of tumors.

p73 is another tumor suppressor gene, which was identified in 1997 by Kaghad *et al*(17) and is considered to regulate cell growth and induce a cell-cycle blockade and cell apoptosis. Given the functional similarity between p53 and p73, it is not unreasonable to suppose that p73 may also be important for the inhibition of cancer development. It has been reported that WWOX interacts with p73 via its first WW domain and promotes the expression of p73 (18). The tyrosine kinase, Src, phosphorylates WWOX at tyrosine 33 in the first WW domain and enhances its binding to p73. Furthermore, the high WWOX expression level boosts the expression of p73 and triggers the redistribution of nuclear p73 to the cytoplasm, resulting in the promotion of cell apoptosis (18). Therefore, the loss of WWOX expression may decrease or suppress the functional role of p73 in apoptosis, which may lead to the occurrence of tumors. All these findings demonstrate that WWOX, FHIT and p73 have important roles in the pathogenesis of tumors and that WWOX may cooperate with FHIT and p73 in the development of tumors.

Epigenetic changes contribute greatly to leukemia development (19). DNA methylation is a well-studied mechanism in epigenetics. The hypermethylation of numerous genes has been detected in various types of tumors and hematological neoplasms (20). Previous studies have shown that DNA methylation is the most commonly detected alteration in ALL (21,22). DNA methylation often results in the silencing of tumor suppressor genes, although the gene sequence may not have been changed. This mechanism has been identified as leading to the loss of function of numerous tumor suppressor genes in various types of tumor cells (23). To clarify how WWOX, FHIT and p73 are involved in the development and progression of ALL, the present study examined the methylation status and mRNA expression of these three tumor suppressor genes in ALL.

## Materials and methods

### Patients and samples

Bone marrow (BM) samples from 112 ALL patients were collected from the Hematology Department of The First Affiliated Hospital of Guangxi Medical University (Nanning, Guangxi, China). In addition to the 112 ALL patients (68 male and 44 female) of the experimental group, 35 non-ALL patients (19 male and 16 female) were included in the study as the control group. According to the diagnosis status of the ALL patient group, there were 72 newly diagnosed patients (NDPs), 26 complete remission patients (CRPs) and 14 relapsed patients (RPs). The diagnosis of ALL was based on morphology, histopathology, expression of leukocyte differentiation antigens and the French American British (FAB) classification. Prior consent from was obtained the patients for the use of these clinical materials for research purposes and study approval was granted from the Ethics Committee of The First Affiliated Hospital of Guangxi Medical University.

### WWOX, FHIT and p73 transcript expression

Total RNA was extracted from the BM samples using TRIzol (Tiangen Inc., Beijing, China), according to the manufacturer's standard instructions. cDNA was synthesized from each RNA using a random primer and Moloney murine leukemia virus reverse transcriptase (Super-Script II; Gibco BRL, Gaithersburg, ND, USA), according to the manufacturer's instructions. The PCR primers used for the RT-PCR in the study are shown in Table I. β-actin expression was used as a control. PCR was performed in a final volume of 25 μl, containing 1 μl cDNA, 2.5 μl 10X PCR buffer, 2 μl dNTPs, 1 μl forward primer, 1 μl reverse primer, 0.15 μl Taq DNA polymerase and 11.3 μl sterilized DEPC water. RT-PCR was conducted in a PE480 DNA Thermal Cycler. The amplification condition were as follows: 94°C for 45 sec, followed by 60°C (WWOX), 58°C (p73) or 60°C (FHIT) for 30 sec and then 72°C for 45 sec, for 35 cycles. The amplification products were analyzed with 2.0% agarose gel electrophoresis. The electrophoresis results were observed with a gel imaging system and photographs of the gels were taken.

### Analysis of WWOX, FHIT and p73 methylation

The methylation status of the first exon region of p73 and the promoter region of WWOX and FHIT was studied. High molecular weight DNA was prepared from cell samples using standard methods, and bisulphate modification of genomic DNA was performed as previously reported (24). The PCR primers used for the methylation-specific PCR (MS-PCR) in the study are also shown in Table I. The 20-μl reaction mixture contained 2.5 μl 10X PCR buffer, 2 μl dNTPs, 1 μl forward primer, 1 μl reverse primer, 2 μl Taq DNA polymerase, 2 μl modified DNA and 11.3 μl sterilized double distilled water. Amplification was performed with a Thermo PCR machine (Thermo Fisher Scientific, Waltham, MA, USA). The MS-PCR reaction conditions for were as follows: Methylated WWOX, 94°C for 30 sec, 46°C for 30 sec and 72°C for 30 sec, for 40 cycles; unmethylated WWOX, 94°C for 30 sec, 52°C for 30 sec and 72°C for 30 sec, for 40 cycles; methylated FHIT, 94°C for 30 sec, 51°C for 30 sec and 72°C for 30 sec, for 40 cycles; unmethylated FHIT, 94°C for 30 sec, 53°C for 30 sec and 72°C for 30 sec, for 40 cycles; methylated p73, 94°C for 30 sec, 51°C for 30 sec and 72°C for 30 sec, for 40 cycles; and unmethylated p73, 94°C for 30 sec, 53°C for 30 sec and 72°C for 30 sec, for 40 cycles. DNA treated *in vitro* with SSSI methyltransferase and untreated DNA was used as the positive control for the methylated and unmethylated templates, respectively. Negative control samples without DNA were included for each set of PCR. The amplification products were analyzed with 2.0% agarose gel electrophoresis, then the electrophoresis results were observed with a gel imaging system and images were captured. The presence of only the methylated amplification product was considered to indicate complete methylation, while the presence of the methylation and unmethylated amplification product indicated partial methylation. All MSP was performed using the EZ DNA Methylation-Gold™ kit (Zymo Research, Irvine, CA, USA).

### Statistical analysis

All statistical analyses were performed using SPSS 13.0 software. The χ^2^ test was used to analyze the associations in the mRNA expression and methylation frequency of WWOX, FHIT and p73 between all the patients. Spearman's rank correlation test was used to analyze possible linear correlations between the expression of all three genes, the gene methylation status and the possible association between gene expression and methylation frequency. Fisher's exact test was used in data numeration. P<0.05 (confidence >95%) was considered to indicate a statistically significant difference.

## Results

### WWOX, FHIT and p73 transcript expression

**Figure 1:**
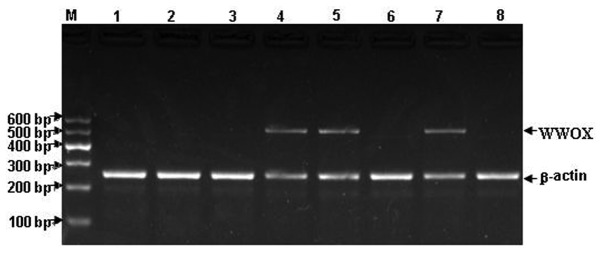
WW-domain oxidoreductase (WWOX) mRNA expression in acute lymphoblastic leukemia (ALL) and control samples. WWOX mRNA expression was observed in samples 4, 6 and 7. M, DNA marker; 1–6 and 8, ALL samples; 7, control samples.

**Figure 2:**
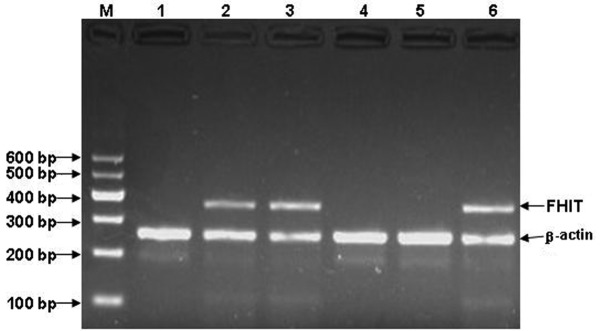
Fragile histidine triad (FHIT) mRNA expression in acute lymphoblastic leukemia (ALL) and control samples. FHIT mRNA expression was observed in samples 2, 3 and 6. M, DNA marker; 1–5, ALL samples; 6, control samples.

**Figure 3:**
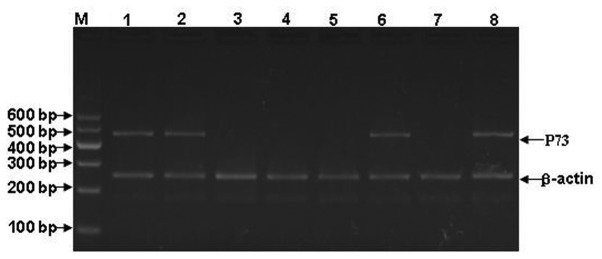
p73 mRNA expression in acute lymphoblastic leukemia (ALL) and control samples. p73 mRNA expression was observed in samples 1, 2, 6 and 8. M, DNA marker; 1–7, ALL samples; 8, control samples.

**Figure 4:**
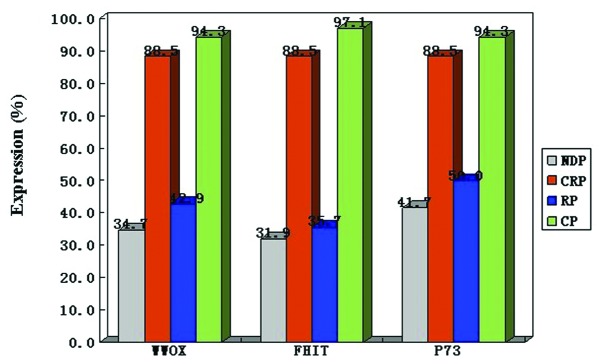
mRNA expression levels of WW-domain oxidoreductase (WWOX), fragile histidine triad (FHIT) and p73 in acute lymphoblastic leukemia (ALL) and control samples. Significantly lower mRNA expression of WWOX, FHIT and p73 was observed in the NDP and RP groups (P<0.05). NDP, newly diagnosed patients; CRP, complete remission patients; RP, relapsed patients; CP, control patients.

As the expression of WWOX, FHIT and p73 mRNA is too low to detect routinely by RNA blot analysis of small samples, RT-PCR amplification was performed to detect WWOX, FHIT and p73 expression, as previously described for leukemia and other tumors (9,25,26). As shown in Table II, the expression of WWOX was not detected in 58 of 112 (51.8%) ALL patients. However, only two out of 35 (5.7%) controls did not exhibit the expression of WWOX. Similar results were observed for FHIT and p73. Significant differences were observed in WWOX, FHIT and p73 expression between the ALL patients and controls (P<0.05). Considering that the results may differ in different stages of ALL, the ALL patients were further classified by their diagnosis status of NDP, CRP and RP. As a result, only 25 of 72 (34.7%) NDPs and 6 of 14 (42.9%) RPs exhibited WWOX expression. However, 23 of 26 (88.5%) CRPs exhibited WWOX expression. Similar results were detected for FHIT and p73. Significant differences were observed in WWOX, FHIT and p73 expression levels between NDPs and CRPs (P<0.05) and RPs and CRPs (P<0.05), but not between NDPs and RPs (P>0.05). The electrophoresis gels of WWOX, FHIT and p73 expression are shown in Figs. 1–3. The mRNA expression of WWOX, FHIT and p73 in the patients with ALL is shown in Fig. 4.

### Methylation status of WWOX, FHIT and p73

**Figure 5:**
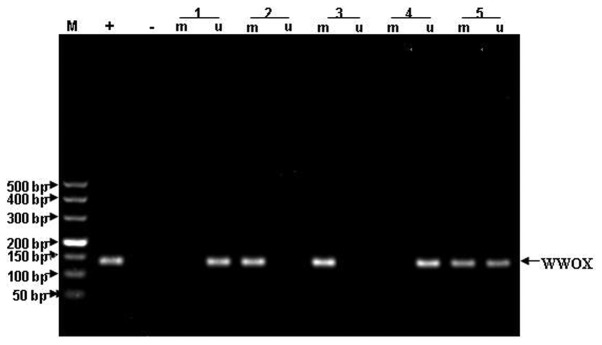
Methylation results of WW-domain oxidoreductase (WWOX) in acute lymphoblastic leukemia (ALL) and control samples according to methylation-specific (MS)-PCR analysis. Samples 2 and 3 were methylated, while 5 was partly methylated. M, DNA marker; +, positive control; −, negative control; m, methylated; u, unmethylated; 1, control sample; 2–5, ALL samples.

**Figure 6:**
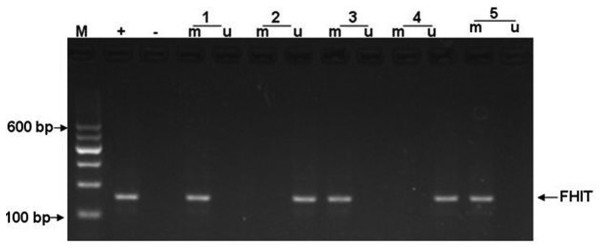
Methylation results of fragile histidine triad (FHIT) in acute lymphoblastic leukemia (ALL) and control samples according to methylation-specific (MS)-PCR analysis. Samples 1, 3 and 5 were methylated. M, DNA marker; +, positive control; −, negative control; m, methylated; u, unmethylated; 2, control sample; 1 and 3–5, ALL samples.

**Figure 7:**
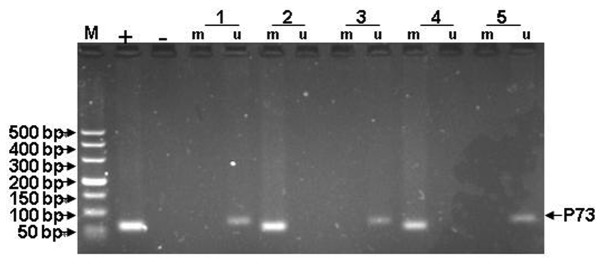
Methylation results of p73 in acute lymphoblastic leukemia (ALL) and control samples according to methylation-specific (MS)-PCR analysis. Samples 2 and 4 were methylated. M, DNA marker; +, positive control; −, negative control; m, methylated; u, unmethylated; 1 control sample; 2–5 ALL samples.

**Figure 8:**
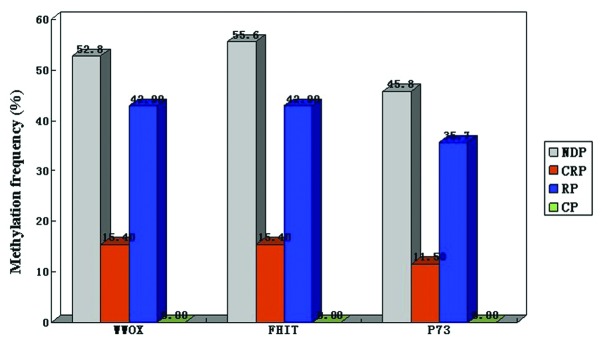
Methylation status of WW-domain oxidoreductase (WWOX), fragile histidine triad (FHIT) and p73 in acute lymphoblastic leukemia (ALL) and control samples. A significantly higher methylation frequency was observed for WWOX, FHIT and p73 in the ALL group compared with the control samples (P<0.05). Furthermore, a significantly lower methylation frequency was observed for WWOX, FHIT and p73 in the CRP group compared with the NDP group (P<0.05). NDP, newly diagnosed patients; CRP, complete remission patients; RP, relapsed patients; CP, control patients.

As described in Table III, although 50 of 112 (44.6%) patients exhibited methylation in the WWOX promoter region, no methylation was detected in the same areas of the control samples. Parallel results were observed in the promoter region of FHIT and the first exon region of p73. Significant differences were observed for the WWOX, FHIT and p73 methylation frequency between the ALL patients and controls (P<0.05). Furthermore, the WWOX, FHIT and p73 methylation frequency was examined in the ALL patients classified by their diagnosis status of NDP, CRP and RP. Consequently, 38 of 72 (52.8%) NDPs, 4 of 26 (15.4%) CRPs and 6 of 14 (42.9%) RPs were observed to have methylation in the WWOX promoter region. Significant differences concerning the methylation frequency of the WWOX promoter region were observed, but only for between NDP and CRP (P<0.05) and not between NDP and RP (P>0.05) or CRP and RP (P>0.05). Consistent results were also observed in the FHIT promoter region and the first exon region of p73. The gel images of WWOX, FHIT and p73 methylation are shown in Figs. 5–7. The methylation status of WWOX, FHIT and p73 in the ALL patients is shown in Fig. 8.

### Associations between methylation status and mRNA expression

Spearman's rank correlation test showed that the mRNA expression levels of WWOX, FHIT and p73 were inversely correlated with the methylation frequency in the ALL patients. The correlation coefficients were −0.661 (P<0.05) for WWOX, −0.685 (P<0.05) for FHIT and −0.536 (P<0.05) for p73.

### Associations between WWOX, FHIT and p73

Spearman's rank correlation test was also used to analyze the associations between the three genes in ALL. The correlation coefficient of mRNA expression between WWOX and FHIT was 0.590 (P<0.05) and between WWOX and p73 was 0.520 (P<0.05). However, no significant correlation was observed in the methylation frequency between WWOX and FHIT (P>0.05) or WWOX and p73 (P>0.05).

## Discussion

In previous studies, the loss or reduction of WWOX and FHIT expression has been shown to be an important step in the initiation of tumorigenesis in a variety of tumors, including those in the breasts, lungs, esophagus, kidneys and cervix (11–16). Furthermore, it has also been reported that altered or absent WWOX expression may suppress the functional role of p73 in apoptosis, leading to an increased possibility of tumor occurrence (18). The aim of the present study was to determine whether there was a similar phenomenon in ALL cases. To the best of our knowledge, this is the first study to focus on such a subject.

In the present study, the mRNA expression of WWOX, FHIT and p73 was analyzed in ALL. As in other tumors, a reduced expression of WWOX, FHIT and p73 was observed in ALL. The present study showed that the expression levels of WWOX and FHIT were altered concordantly in ALL, and that the frequency of WWOX expression, as determined by RT-PCR amplification of mRNA, was almost the same as that of FHIT (48.2 vs. 42.9%). In previous studies, the reported frequency of FHIT alterations in ALL has varied; the expression of FHIT mRNA or protein has been reported to be altered in 20–70% of cases (27–30). A previous study investigating p73 expression in ALL revealed relatively small differences, as p73 expression in ALL differed by no more than 40% (31). It reported that p73 expression is exhibited in 61–100% of ALL (31); p73 expression was detected in 55.4% of ALL in the present study, which was slightly less than previous results. The results of previous studies approximated to those of the present study, which may to a certain extent provide evidence for and support the conclusions of this study. In the present study, the cases that showed WWOX alterations also showed alterations to FHIT and exhibited almost the same mRNA expression. These observations suggest that WWOX and FHIT may concordantly affect the progression of ALL. Moreover, a number of authors have shown that the suppressed transcription of WWOX and FHIT is associated with the more advanced stages in various types of cancer, including breast (32,35), non-small cell lung (33) and ovarian (34) cancers. The present study demonstrated that a relatively low expression of WWOX and FHIT correlates with NDP and RP diagnosis statuses in ALL patients, supporting the hypothesis that the occurrence of ALL is a progressive and multi-staged process, similar to that of other tumors. The expression of p73 in ALL was not significantly higher than that of WWOX (48.2 vs. 55.4%), although WWOX and p73 expression was significantly lower compared with the controls, indicating that the loss of WWOX expression may inhibit the apoptotic role of p73 and cause ALL to occur.

Despite the frequent suppression of WWOX, FHIT and p73 expression in numerous cancers, complete gene inactivation by the deletion of one allele and a second mutation or homozygous deletion is extremely rare (36). Based on these observations, it has been postulated that the inactivation of WWOX, FHIT and p73 is driven by hemizygous deletions or methylation status. However, loss of heterozygosity (LOH) and somatic mutations are rare in hematological malignancies (37). Epigenetic changes are of great importance to leukemia development (19). Previous studies have shown that DNA methylation is the most commonly detected alteration in AL (21,22). Therefore, the methylation status was investigated as the inactivating mechanism of WWOX, FHIT and p73 in ALL in the present study. At present, FHIT and WWOX promoter methylation associated with the loss of gene expression has been reported for various types of cancers, including lung, breast, esophageal and bladder cancer (38). Similarly, a higher methylation frequency of WWOX, FHIT and p73 was shown in ALL in the present study. Furthermore, the NDP and RP groups also showed the higher methylation frequency of the three genes in ALL, suggesting that the methylation of WWOX, FHIT and p73 in ALL was accumulated through the progression of the disease. It has been reported that 30% of ALL cases have the methylated p73 status (39), which is close to the 37.5% methylation frequency of p73 in the present study. Previous studies concerning the frequency of FHIT methylation in ALL have differed, ranging between 13.8 and 67.0% (40–43). The present study demonstrated 46.4% FHIT methylation in the ALL cases. The difference is possibly due to the diagnosis time for ALL, which leads to the alteration of FHIT methylation frequency, as numerous studies have demonstrated that FHIT methylation is an accumulated process and different diagnosis times exhibit different methylation frequencies (40–42). Research on the methylation of the WWOX promoter region has mainly focused on a few malignant tumors, such as stomach cancer (44), glioblastoma (45) and lung cancer (46). To date, no study has yet been reported on the methylation of the WWOX promoter region in ALL.

Iliopoulos *et al*(38) studied the WWOX promoter methylation status in pancreatic cancer and reported methylation in region −148 to −37, respective to the transcription initiation site (+1), in pancreatic adenocarcinomas; the WWOX promoter methylation status was associated with its expression and following treatment with 5-aza-deoxycytidine in Hs766T cells, WWOX was expressed again. Maeda *et al*(44) reported that 24 of 73 (32.9%) stomach cancer cases that showed absent WWOX expression were highly methylated. These studies have suggested that WWOX methylation causes the loss of or decreased expression of WWOX. The present study showed that the mRNA expression of WWOX, FHIT and p73 was reversely correlated with the methylation of WWOX, FHIT and p73 in ALL, indicating that the highly methylated status of WWOX, FHIT and p73 leads to silencing of the expression of these genes, which may result in the loss of gene transcriptions and promote the occurrence and development of ALL. Notably, significant differences were detected in the mRNA expression of WWOX, FHIT and p73 between the RP and CRP groups with ALL. However, no significant difference was detected in the methylation frequency of the three genes between the RP and CRP groups. We suggest that this confusing phenomenon may be caused by the small sample size of the RP group, as only 14 RPs were enrolled in this study.

The present study observed that the mRNA expression of WWOX was positively correlated with not only FHIT, but also p73, suggesting that to a certain extent, the decrease in the transcription of p73 and FHIT may interact with the reduction of WWOX transcription and further promote the occurrence and development of ALL. However, the present study did not reveal any associations in the methylation status among WWOX, FHIT and p73. It may be inferred that WWOX methylation does not affect the methylation of FHIT and p73.

In summary, the methylation status of WWOX, FHIT and p73 may lead to the silencing of gene expression, promoting the occurrence and development of ALL. Detecting the mRNA expression and methylation status of WWOX, FHIT and p73 may aid in the development of future treatment approaches for ALL.

## Figures and Tables

**Table I:** Prime sequence for RT-PCR and MS-PCR.

Gene primer	Primer sequence (5′→3′)	PCR product size, bp
β-actin-F	AACAAGATGAGATTGGCA	251
β-actin-R	AGTGGGGTGGCTTTTAGGAT	
rtpcr-WWOX-F	AATCACACCGAGGAGAAGAC	536
rtpcr-WWOX-R	AAAGTTGCTGCGTTGCACAC	
rtpcr-FHIT-F	ATGTCGTTCAGATTTGGCCAAC	340
rtpcr-FHIT-R	TCATAGATGCTGTCATTCCTGT	
rtpcr-p73-F	CCCACCACTTTGAGGTCACT	461
rtpcr-p73-R	CAGGGTGATGATGATGAGGA	
WWOX-MF	AGGATTGGTTAGAATAACGC	193
WWOX-MR	AAAATACCTAAAAAATCGCG	
WWOX-UF	TGTAGGATTGGTTAGAATAATGT	193
WWOX-UR	AAAAATACCTAAAAAATCACACT	
FHIT-MF	TTTTCGTTTTTGTTTTTAGATAAGC	157
FHIT-MR	AAAAATATACCCACTAAATAACCGC	
FHIT-UF	TGGTTTTTGTTTTTGTTTTTAGATAAGT	159
FHIT-UR	AAAATATACCCACTAAATAACCACC	
p73-MF	GGACGTAGCGAAATCGGGGTTC	68
p73-MR	ACCCCGAACATCGACGTCCG	
p73-UF	AGGGGATGTAGTGAAATTGGGGTTT	77
p73-UR	ATCACAACCCCAAACATCAACATCCA	

MS, methylation-specific; F, forward primer; R, reverse primer; M, methylated; U, unmethylated; WWOX, WW-domain oxidoreductase; FHIT, fragile histidine triad.

**Table II:** WWOX, FHIT and p73 expression in ALL and controls.

		WWOX		FHIT		p73	
				
Diagnosis	n	Detected	Not detected	Detected	Not detected	Detected	Not detected
ALL	112	54	58	48	64	62	50
NDP	72	25	47	23	49	30	42
CRP	26	23	3	23	3	23	3
RP	14	6	8	5	9	7	7
Controls	35	33	2	34	1	33	2

ALL, acute lymphoblastic leukemia; WWOX, WW-domain oxidoreductase; FHIT, fragile histidine triad; NDP, newly diagnosed patients; CRP, complete remission patients; RP, relapsed patients.

**Table III:** WWOX, FHIT and p73 methylation frequency in ALL and controls.

		WWOX		FHIT		p73	
				
Diagnosis	n	Methylated	Unmethylated	Methylated	Unmethylated	Methylated	Unmethylated
ALL	112	50	62	52	60	42	70
NDP	72	38	34	40	32	33	39
CRP	26	4	22	4	22	3	23
RP	14	6	8	6	8	5	9
Controls	35	0	35	0	35	0	35

ALL, acute lymphoblastic leukemia; WWOX, WW-domain oxidoreductase; FHIT, fragile histidine triad; NDP, newly diagnosed patients; CRP, complete remission patients; RP, relapsed patients.

## References

[1] Pui CH, Relling MV, Downing JR (2004). Acute lymphoblastic leukemia. N Engl J Med.

[2] Iughetti L, Bruzzi P, Predieri B, Paolucci P (2012). Obesity in patients with acute lymphoblastic leukemia in childhood. Ital J Pediatr.

[3] Dalmasso P, Pastore G, Zuccolo L (2005). Temporal trends in the incidence of childhood leukemia, lymphomas and solid tumors in north-west Italy, 1967–2001. A report of the Childhood Cancer Registry of Piedmont. Haematologica.

[4] Redaelli A, Laskin BL, Stephens JM, Botteman MF, Pashos CL (2005). A systematic literature review of the clinical and epidemiological burden of acute lymphoblastic leukaemia (ALL). Eur J Cancer Care (Engl).

[5] Smith MA, Seibel NL, Altekruse SF (2010). Outcomes for children and adolescents with cancer: challenges for the twenty-first century. J Clin Oncol.

[6] Turner BC, Ottey M, Zmonjic DB (2002). The fragile histidine triad/common chromosome fragile site 3B locus and repair-deficient cancer. Cancer Res.

[7] Nunez MI, Ludes-Meyer J, Abba MC (2005). Frequent loss of WWOX expression in breast cancer: correlation with estrogen receptor status. Breast Cancer Res Treat.

[8] Ilsley JL, Sudol M, Winder SJ (2002). The WW domain: linking cell signaling to the membrane cytoskeleton. Cell Signal.

[9] Ohta M, Inoue H, Cotticelli MG (1996). The FHIT gene, spanning the chromosome 3p14.2 fragile site and renal carcinoma-associated t (3;8) breakpoint, is abnormal in digestive tract cancers. Cell.

[10] Bednarek AK, Laflin KJ, Daniel RL, Liao Q, Hawkins KA, Aldaz CM (2000). WWOX, a novel WW domain-containing protein mapping to human chromosome 16q2.33–2.41, a region frequently affected in breast cancer. Cancer Res.

[11] Aqeilan RI, Croce CM (2007). WWOX in biological control and tumorigenesis. J Cell Physiol.

[12] Sozzi G, Tornielli S, Tagliabue E (1997). Absence of Fhit protein in primary lung tumor and cell lines with FHIT gene abnormalities. Cancer Res.

[13] Tseng JE, Kemp BL, Khuri FR (1999). Loss of Fhit is frequent in stage I non-small cell lung cancer and in the lungs of chronic smokers. Cancer Res.

[14] Mori M, Mimori K, Shiraishi T (2000). Altered expression of Fhit in carcinoma and precarcinomatous lesions of the esophagus. Cancer Res.

[15] Connolly DC, Greenspan DL, Wu R (2000). Loss of Fhit expression in invasive cervical carcinomas and intraepithelial lesions associated with invasive disease. Clin Cancer Res.

[16] Guler G, Uner A, Guler N (2005). Concordant loss of fragile gene expression early in breast cancer development. Pathol Int.

[17] Kaghad M, Bonnet H, Yang A (1997). Monoallelically expressed gene related to p73 at 1p36, a region frequently deleted in neuroblastoma and other human cancers. Cell.

[18] Aqeilan RI, Pekarsky Y, Herrero JJ (2004). Functional association between Wwox tumor suppressor protein and p73, a p53 homolog. Proc Natl Acad Sci USA.

[19] Agrawal S, Unterberg M, Koschmieder S (2007). DNA methylation of tumor suppressor genes in clinical remission predicts the relapse risk in acute myeloid leukemia. Cancer Res.

[20] Esteller M (2002). CpG island hypermethylation and tumor suppressor genes: A booming present, a brighter future. Oncogene.

[21] Garcia-Manero G, Daniel J, Smith TL (2002). DNA methylation of multiple promoter-associated CpG islands in adult acute lymphocytic leukemia. Clin Cancer Res.

[22] Roman-Gomez J, Jimenez-Velasco A, Castillejo JA (2004). Promoter hypermethylation of cancer-related genes: A strong independent prognostic factor in acute lymphoblastic leukemia. Blood.

[23] Nephew KP, Huang TH (2003). Epigenetic gene silencing in cancer initiation and progression. Cancer Lett.

[24] Herman JG, Graff JR, Myöhänen S, Nelkin BD, Baylin SB (1996). Methylation-specific PCR: a novel PCR assay for methylation status of CpG islands. Proc Natl Acad Sci USA.

[25] Kuroki T, Trapasso F, Shiraishi T (2002). Genetic alterations of the tumor suppressor gene WWOX in esophageal squamous cell carcinoma. Cancer Res.

[26] Yendamuri S, Kuroki T, Trapasso F (2003). WW domain containing oxidoreductase gene expression is altered in non-small cell lung cancer. Cancer Res.

[27] Lin PM, Liu TC, Chang JG, Chen TP, Lin SF (1997). Aberrant FHIT transcripts in acute myeloid leukemia. Br J Haematol.

[28] Iwai T, Yokota S, Nakao M (1998). Frequent aberration of FHIT gene expression in acute leukemias. Cancer Res.

[29] Albitar M, Manshouri T, Gidel C (2001). Clinical significance of fragile histidine triad gene expression in adult acute lymphoblastic leukemia. Leuk Res.

[30] Hallas C, Albitar M, Letofsky J, Keating MJ, Huebner K, Croce CM (1999). Loss of FHIT expression in acute lymphoblastic leukemia. Clin Cancer Res.

[31] Pluta A, Nyman U, Joseph B, Robak T, Zhivotovsky B, Smolewski P (2006). The role of p73 in hematological malignancies. Leukemia.

[32] Campiglio M, Pekarsky Y, Menard S, Tagliabue E, Pilotti S, Croce CM (1999). FHIT loss of function in human primary breast cancer correlates with advanced stage of the disease. Cancer Res.

[33] Donati V, Fontanini G, Dell'Omodarme M (2007). WWOX expression in different histologic types and subtypes of non-small cell lung cancer. Clin Cancer Res.

[34] Nunez MI, Rosen DG, Ludes-Meyers JH (2005). WWOX protein expression varies among ovarian carcinoma histotypes and correlates with less favorable outcome. BMC Cancer.

[35] Płuciennik E, Kusińska R, Potemski P, Kubiak R, Kordek R, Bednarek AK (2006). WWOX - the FRA16D cancer gene: expression correlation with breast cancer progression and prognosis. Eur J Surg Oncol.

[36] Alsop AE, Taylor K, Zhang J, Gabra H, Paige AJ, Edwards PA (2008). Homozygous deletions may be markers of nearby heterozygous mutations: the complex deletion at FRA16D in the HCT116 colon cancer cell line removes exons of WWOX. Genes Chromosomes Cancer.

[37] Iwai M, Kiyoi H, Ozeki K (2005). Expression and methylation status of the FHIT gene in acute myeloid leukemia and myelodysplastic syndrome. Leukemia.

[38] Iliopoulos D, Guler G, Han SY (2006). Roles of FHIT and WWOX fragile genes in cancer. Cancer Lett.

[39] Corn PG, Kuerbitz SJ, van Noesel MM (1999). Transcriptional silencing of the p73 gene in acute lymphoblastic leukemia and Burkitt's lymphoma is associated with 5′ CpG island methylation. Cancer Res.

[40] Zheng S, Ma X, Zhang L (2004). Hypermethylation of the 5′ CpG island of the FHIT gene is associated with hyperdiploid and translocation-negative subtypes of pediatric leukemia. Cancer Res.

[41] Iwai M, Kiyoi H, Ozeki K (2005). Expression and methylation status of the FHIT gene in acute myeloid leukemia and myelodysplastic syndrome. Leukemia.

[42] Yang Y, Takeuchi S, Hofmann WK (2006). Aberrant methylation in promoter-associated CpG islands of multiple genes in acute lymphoblastic leukemia. Leuk Res.

[43] Paulsson K, An Q, Moorman AV (2009). Methylation of tumour suppressor gene promoters in the presence and absence of transcriptional silencing in high hyperdiploid acute lymphoblastic leukaemia. Br J Haematol.

[44] Maeda N, Semba S, Nakayama S, Yanagihara K, Yokozaki H (2010). Loss of WW domain-containing oxidoreductase expression in the progression and development of gastric carcinoma: clinical and histopathologic correlations. Virchows Arch.

[45] Kosla K, Pluciennik E, Kurzyk A (2011). Molecular analysis of WWOX expression correlation with proliferation and apoptosis in glioblastoma multiforme. J Neurooncol.

[46] Baykara O, Demirkaya A, Kaynak K, Tanju S, Toker A, Buyru N (2010). WWOX gene may contribute to progression of non-small-cell lung cancer (NSCLC). Tumour Biol.

